# Lymphocutaneous sporotrichosis: A case report from southeastern China and review of the literature

**DOI:** 10.1097/MD.0000000000050812

**Published:** 2026-09-25

**Authors:** Xiaoqing Wang, Jing Bian, Jianping Xu, Yaqing Li, Shujuan Ji, Li Qin, Qingsong Meng

**Affiliations:** a Department of Infectious Disease, Hebei General Hospital, Shijiazhuang, China; b Department of Infectious Disease, Sir Run Run Shaw Hospital, Zhejiang University School of Medicine, Hangzhou, China; c Department of Urology Surgery, The Second Hospital of Hebei Medical University, Shijiazhuang, Hebei, China.

**Keywords:** itraconazole, lymphocutaneous sporotrichosis, metagenomic next-generation sequencing (mNGS), *Sporothrix globosa*

## Abstract

**Rationale:**

Sporotrichosis is a dermatomycosis caused by the dimorphic fungus *Sporothrix schenckii* sensu lato. The traditional gold standard for the definitive diagnosis of the disease is fungal culture. Our case describes the patient diagnosed lymphocutaneous sporotrichosis through the metagenomic next-generation sequencing (mNGS) quickly.

**Patient concerns:**

A 53-year-old male presented with protracted multiple ulcerated lesions. The pathological analysis of his skin biopsy specimen was inflammatory reaction, routine hematological and biochemistry examinations, the imaging of heart, lung and stomach were normal.

**Diagnoses:**

The wounds were received debridement in our ward. *Sporothrix schenckii* was identified by the culture and the mNGS of the wound pus.

**Interventions:**

Following definitive diagnosis, oral itraconazole was administered to the patient; meanwhile, regular clinical follow-up visits and reevaluations were scheduled for continuous monitoring.

**Outcomes:**

The patient recovered well, and no signs of recurrence were detected during follow-up.

**Lessons:**

This case illustrates that mNGS, as an emerging molecular biological diagnostic technique, holds significant diagnostic value for the rapid identification of sporotrichosis. Timely and accurate diagnosis is essential for achieving favorable clinical outcomes.

## 1. Introduction

Sporotrichosis is a cutaneous fungal infection with a global distribution, occurring preferably in tropical and subtropical regions.^[1]^ First identified in 1898, *Sporothrix schenckii* was once regarded as the exclusive etiologic agent of sporotrichosis. However, with the development of molecular biology techniques, we discovered that *S. schenckii* is a complex of at least 6 important species with distinct biochemical properties.^[2,3]^ *Sporothrix schenckii* species complex can be isolated from saprophytes in soil,decaying wood, hay and even marine animals. The disease is mainly caused by traumatic implantation of the fungus during outdoor activities such as farming, gardening, and animal husbandry.^[4]^ Several individuals were infected through inhalation of pathogens.^[5]^ The disease has several clinical forms, mainly cutaneous with associated lymphadenopathy, uncommon clinical features like pulmonary or disseminated disease mostly arises in immunosuppressed individuals.^[6]^ In China, *Sporothrix globosa* predominates, especially in the northeastern endemic regions.^[7–9]^ Fungal culture is still the primary diagnostic method for sporotrichosis.^[4,5]^ Timely recognition of infectious pathogens remains a critical clinical requirement, and metagenomic next-generation sequencing (mNGS) facilitates precise pathogen identification from clinical specimens. This case attained rapid diagnosis with fungal culture and mNGS, and oral itraconazole achieved complete remission.

## 2. Case presentation

A 53-year-old male living in Ningbo, a city of southeastern China. He visited us on October 14, 2021, with the chief complaint of a 5-month history of slowly enlarging, festering wounds that started on his left thumb, extended to his ipsilateral forearm 2 months later and then to his ipsilateral upper-arm. No fever, chills, headache, and weight loss had occurred. Previous treatments with antibiotics and topical ointments caused no improvement. He had a history of impaired glucose tolerance and hypertension, but no immunodeficiency diseases. He reported an accidental stab by wood chips before the onset of illness.

Physical examination revealed multiple ulcerated nodules along lymphatic tracts on the hand and forearm with chronic inflammatory changes surrounding the ulcers, accompanied by exudate and surrounding tissue hyperplasia (Figs. 1A-C). Debridement of the wounds was performed in our ward. Subsequently, for definitive identification of the pathogen, pus specimen culture and smear examination were conducted on day 1, we also sent pus specimens for mNGS test. We found yeast-like fungi by immunofluorescence staining (Fig. 2A) on day 2. On day 3, the mNGS detected *Sporothrix globosa* with 2652 high-confidence sequence reads that showed 99.0% identity and covered 0.83% of the *Sporothrix globosa* genome (National center for biotechnology information BioProject ID:PRJNA816636)(Fig. 3). Routine hematological and biochemistry investigations, including the interferon-gamma release assay, immunologic tests, acid-fast staining and modified acid-fast staining, Giemsa staining, mycobacterial culture showed normal results, thus ruling out tuberculosis, nocardiosis, cutaneous leishmaniasis and cutaneous nontuberculous mycobacterial infection. The histopathology of previous hospital showed acute and chronic inflammation associated with epithelial hyperplasia and multinucleated cell reactions, making lymphoid-associated tumors less likely. The chronic inflammatory cutaneous lesion in our patient was a fixed traumatic ulcer with sequential subcutaneous nodule development along lymphatic vessels and a poor response to anti-eczema therapies, which differs from eczema featuring recurrent pruritus, symmetric erythema, rare ulcer formation and absent lymphatic dissemination. Fungal culture of purulent exudate was performed on Sabouraud dextrose agar at 25°C for 10 days, yielding numerous dark-brown colonies exhibiting a wrinkled, leathery-velvety surface (Fig. 2B). The isolate was identified as a member of the *Sporothrix schenckii* species complex. On the basis of clinical manifestations and laboratory findings, a diagnosis of lymphocutaneous sporotrichosis was established.

**Figure 1.**
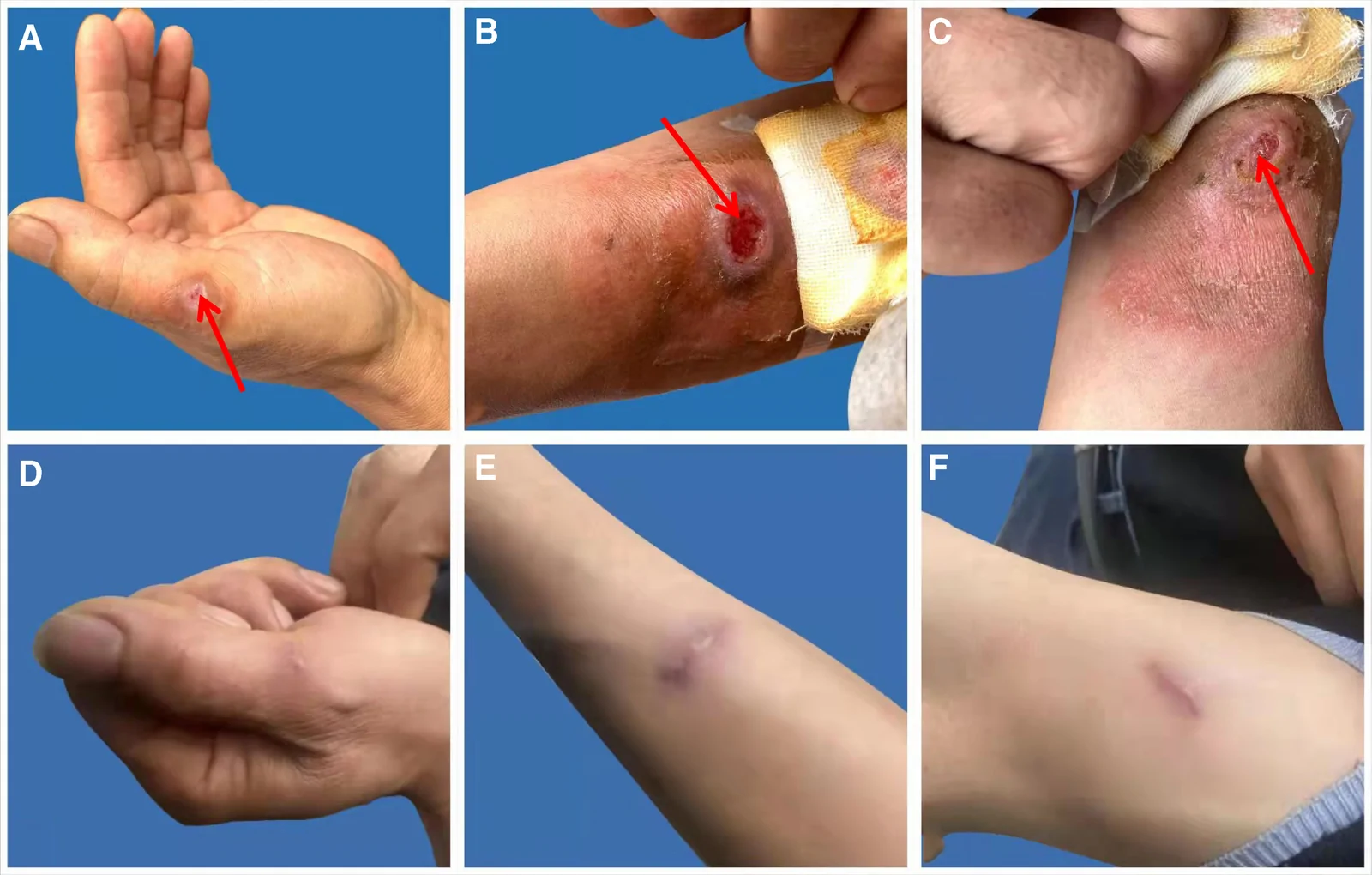
The festering wounds involving the left arm of the patient. (A–C) The wounds, in order, on the left thumb, left forearm, and left upper-arm. (D–F) The lesions, in order, on the left thumb, left forearm, and left upper arm were healed after 3 months of oral itraconazole. Red arrows: biopsy site.

**Figure 2.**
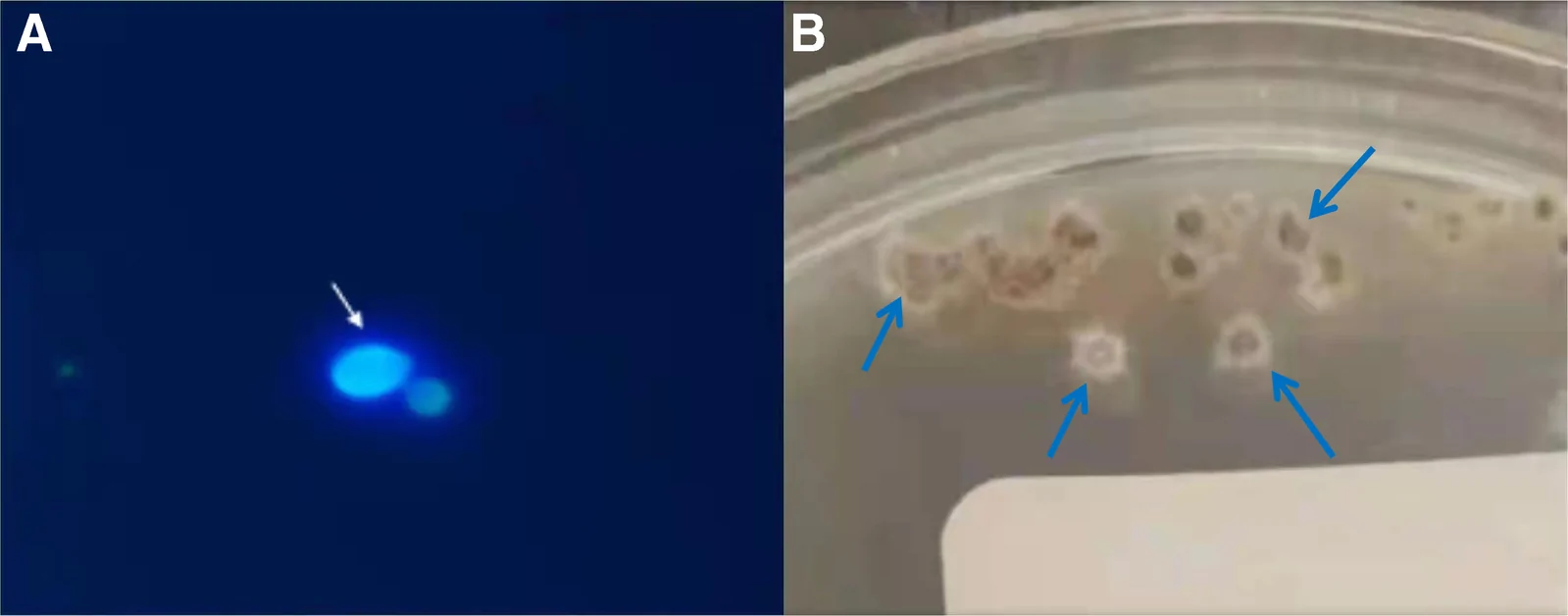
(A) Immunofluorescence staining showing yeast-like fungus (white arrow). (B) Sporothrix schenckii colony on Sabouraud’s glucose agar (SDA) at 25°C (blue arrows).

**Figure 3.**
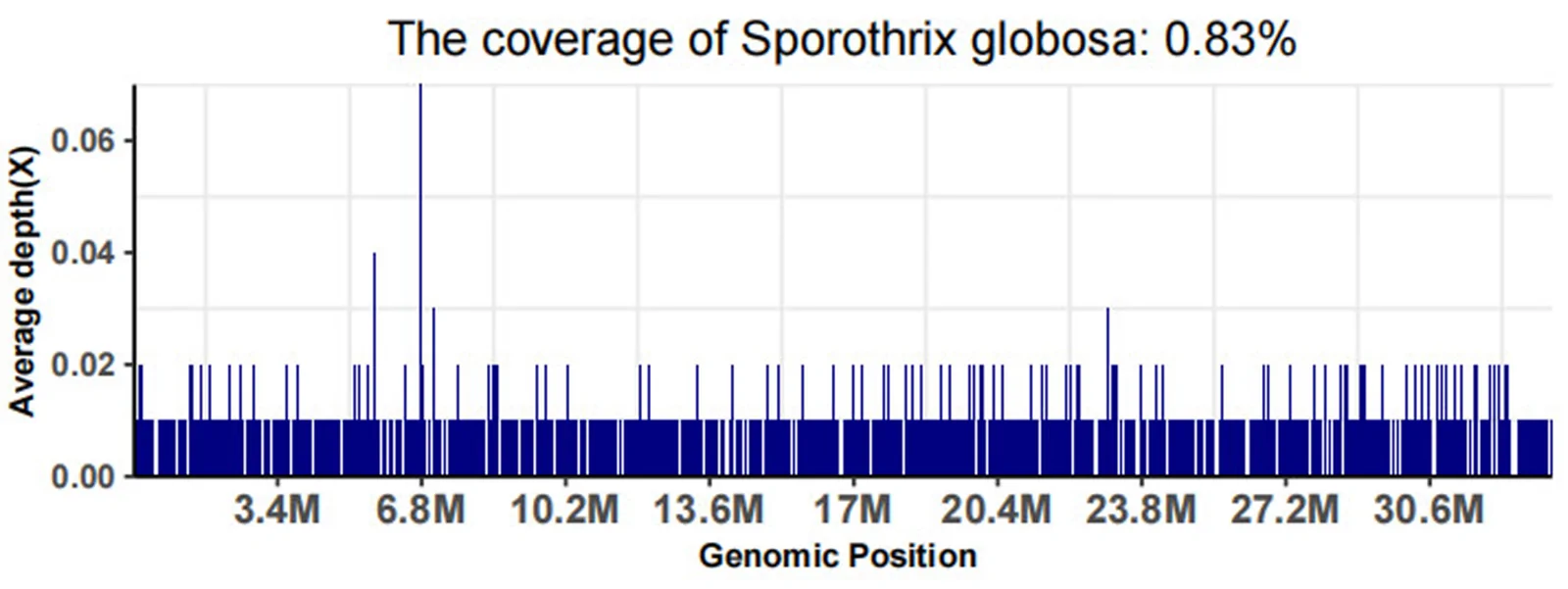
Metagenomic next-generation sequencing result of our patient: A total of 2652 specific Sporothrix globosa sequences that covered 0.83% of the total Sporothrix globosa genome were detected by mNGS in the pus sample of the patient.

The patient started oral itraconazole (200 mg twice daily for 7 days, followed by 200 mg once daily for 3 months). The lesions achieved complete healing without exudate or discharge after 3 months of itraconazole treatment; no adverse drug reactions were noted (Figs. 1D-F). We informed the patient that effective prevention and control strategies involve consistent use of personal protective equipment, such as gloves, long-sleeve clothing and sturdy boots, to prevent traumatic puncture wounds. The patient is currently under regular follow-up.

## 3. Discussion

*Sporothrix schenckii* is a thermodimorphic fungus, exhibiting mycelial growth at 25°C (saprophytic phase) and yeast-like cell proliferation at 35 to 37°C (pathogenic phase).^[10]^ Currently, the *Sporothrix genus* comprises 51 taxa, which are divided into 2 major clades: a clinical clade including *S. brasiliensis, S. schenckii, S. globosa,* and *S. luriei and* an environmental clade consisting of several species complexes (e.g., *S. pallida, S. candida, S. inflata*) along with numerous subtypes.^[11,12]^ The patient of the report was infected by *S. globosa,* which is consistent with the predominant etiological agent of sporotrichosis in China.^[7,8]^ Orofino-Costa et al^[11]^ also reported that *S.globosa* accounts for an estimated 99.3% of human sporotrichosis cases in Asia, particularly in China.

Epidemiological investigations have demonstrated that approximately 10% to 62% of patients trace their infection back to recent penetrating trauma caused by wood splinters, plant thorns, or contaminated plant debris.^[5]^ Accordingly, sporotrichosis is commonly referred to as “rosebush disease” or “gardener’s mycosis.” In our case, the patient was stabbed by wood chips a week before illness. It is consistent with the wood splinter injuries described in case reports by Pérez-López et al^[13]^ from Spain and Schwalb et al^[14]^ from Peru (Table 1). Nevertheless, both cases were caused by *Sporothrix schenckii*. Similar to our patient, lesions in these 2 cases all occurred on the extremities, presenting as multiple ulcerative and nodular lesions distributed along lymphatic vessels. By contrast, the case reported by Verma et al^[15]^ from India (Table 1) featured no history of traumatic inoculation, yet the pathogen isolated was also *Sporothrix schenckii*, with lesions confined to the face. The case documented by Martins et al^[16]^ from Brazil involved a patient with cat exposure, and lesions developed on the upper extremities. *Sporothrix* species was isolated, though exact species subtype identification was not performed.

**Table 1:** Clinical characteristics of published lymphocutaneous sporotrichosis case reports.

Author, yr	Age, sex	Country	Risk & inoculum	Sporothrix sp.	Manifestations	Diagnostic method	Treatment	Outcome
Perez-López et al 2023 ^[13]^	36, M	Spain	Farmer; trauma inoculation	Sporothrix schenckii	Multiple nodules along hand/forearm lymphatic tracts	Culture	ITZ 200 mg twice daily; L-AmB 5 mg/kg/ d for 4 months	Recovered
Schwalb et al 2022^[14]^	58, M	Peru	Farmer; wood splinter inoculation trauma	Sporothrix schenckii	Multiple ulcerated nodules along hand/forearm lymphatic tracts	Culture	ITZ 200 mg daily for 3 months	Recovered
Verma et al 2019^[15]^	14, M	India	None	Sporothrix schenckii	Warty lesions on the face	Culture	ITZ 100mg twice daily; SSKI for 4 months	Recovered
Martins et al 2023 ^[16]^	64, F	Brazil	Contact with cats	Sporothrix species	Confluent ulceration over the upper limb	Culture	ITZ 100 mg twice daily; L-AmB 3 mg/kg/ d for 21 d	Slight functional limitation
Li et al 2025 ^[17]^	49, F	Beijing, China	None	Sporothrix schenckii	Linear nodules on the face	Culture	SSKI and terbinafine	Recovered
Zhang et al 2016 ^[18]^	59, M	Tianjin, China	Diabetes mellitus	Sporothrix globosa	Multiple ulcerated nodules along hand/forearm lymphatic tracts	Culture+molecular identification	ITZ 100 mg daily for 10 months	Recovered
Ruan et al 2026 ^[19]^	68, F	Zhejiang,China	Diabetes mellitus	Sporothrix globosa	Linear nodules on the face	Culture+molecular identification	ITZ for 3 months	Recovered
Ruan et al 2026^[19]^	48, F	Zhejiang,China	None	Sporothrix globosa	Linear nodules on the face	Culture+molecular identification	ITZ for 3 months	Recovered
Shi et al 2024 ^[20]^	34, M	Zhejiang,China	None	Sporothrix species	Linear nodules on the face	Culture	ITZ for 3 months	Recovered
Yu et al 2026 ^[21]^	47, F	Guangzhou,China	None	Sporothrix schenckii	Multiple ulcerated nodules along hand/forearm lymphatic tracts	Culture+molecular identification	ITZ 100 mg daily for 3 months	Recovered

Ages are reported in years, F = female, ITZ = itraconazole, L-AmB = liposomal amphotericin B, M = male, Sporothrix sp. = Sporothrix species, SSKI = saturated solution of potassium iodide.

As documented by Syed et al ^[1]^ the lymphocutaneous subtype accounts for approximately 80% of sporotrichosis cases, consistent with our patient’s presentation. Previously published lymphocutaneous sporotrichosis case reports are summarized in Table 1. Definitive diagnosis of lymphocutaneous sporotrichosis relies on detection of fungal pathogens in tissue via culture, histopathology, or molecular analysis. Reported from northern China, the cases of Li et al^[17]^ and Zhang et al^[18]^ were caused by *Sporothrix schenckii* and *Sporothrix globosa*, with lesions located on the face and extremities respectively.Consistent with our case, cases documented by Ruan et al^[19]^ from Zhejiang Province in southern China were confirmed to be caused by *Sporothrix globosa*. Another case reported by Shi et al^[20]^ lacked clear species identification of *Sporothrix*. All these previously reported patients presented facial lesions, our case presented extremity lesions but shared the same pathogen, *Sporothrix globosa*.Also from southern China, cases documented by Yu et al^[21]^ in Guangzhou shared identical lesion sites (the extremities) with ours, yet the causative pathogen was *Sporothrix schenckii*. The cases of sporotrichosis have been predominantly reported from Northeast China,^[9,22]^ and the region is recognized as an endemic area for this disease. However, sporotrichosis can also be detected in other regions of China. Notably, the patient in our report is from Southeast China and has no travel history to Northeast China. Clinical manifestations of sporotrichosis vary across different regions of China and overseas; therefore, timely and accurate identification of causative *Sporothrix* species is critical for subsequent treatment.

The differential diagnosis of cutaneous lesions includes tuberculosis, *Mycobacterium marinum* or *Mycobacterium kansasii* infection,nocardiosis, bacterial pyoderma, leishmaniasis, chromoblastomycosis, blastomycosis, and even noninfectious diseases.

Currently, mNGS exhibits higher sensitivity than conventional microbiologic tests for etiological diagnosis of infectious diseases, with particular advantages in identifying rare-pathogen infections.^[12,23–26]^ Clinical samples for testing include skin lesion tissues, biopsy specimens, abscess aspirates, pus, synovial fluid, and cerebrospinal fluid, in addition to blood. Currently, fungal culture remains the primary diagnostic method for sporotrichosis. Rueda et al^[27]^ and He et al^[28]^ respectively reported the cases of disseminated cutaneous sporotrichosis diagnosed by fungal culture on day 15 and day 14, which the latter patient was misdiagnosed as histoplasmosis prior to definitive confirmationin. Giuffrida et al^[29]^ also reported the lymphocutaneous sporotrichosis diagnosed by fungal culture. Rojas et al^[30]^ reported the case of pulmonary sporotrichosis diagnosed by fungal culture, which was further confirmed by molecular method. In our case, we rapidly identified the pathogen and its specific subtype via mNGS performed on pus specimens.

Multiple therapeutic options are available for sporotrichosis, including potassium iodide, itraconazole, fluconazole, terbinafine, amphotericin B, and several physical therapeutic modalities. The choice of treatment depends on both the clinical presentation and the immune status of the patient.^[14]^ Itraconazole is the preferred drug for less severe infections. Fixed cutaneous or lymphocutaneous sporotrichosis should be treated for 2 to 4 weeks after lesion resolution, with a total duration of 3 to 6 months.^[11,14]^ This regimen was consistent with the treatment administered in our case.

## 4. Conclusion

In conclusion, for patients with chronic recurrent ulcerative skin lesions, the possibility of special bacterial infections should be taken into consideration by clinicians. Traditional body fluid culture remains the gold standard for the diagnosis of such infections, whereas modern diagnostic techniques, such as mNGS assays, can markedly improve the efficiency and accuracy of diagnostic identification.

## Acknowledgments

We thank the patient for agreeing to let us report on this case report.

## Author contributions

**Formal analysis:** Xiaoqing Wang.

**Data curation:** Jianping Xu, Yaqing Li.

**Resources:** Li Qin.

**Writing – original draft:** Xiaoqing Wang, Jing Bian, Qingsong Meng.

**Writing – review & editing:** Xiaoqing Wang, Shujuan Ji, Qingsong Meng.
